# Magnetic properties of ball-milled SrFe_12_O_19_ particles consolidated by Spark-Plasma Sintering

**DOI:** 10.1038/srep14112

**Published:** 2015-09-15

**Authors:** Marian Stingaciu, Martin Topole, Paul McGuiness, Mogens Christensen

**Affiliations:** 1 Center for Materials Crystallography, Department of Chemistry and iNANO, Aarhus University, DK-8000 Aarhus C, Denmark; 2 Department for Nanostructured Materials, Jožef Stefan Institute, SI-1000 Ljubljana, Slovenia

## Abstract

The room-temperature magnetic properties of ball-milled strontium hexaferrite particles consolidated by spark-plasma sintering are strongly influenced by the milling time. Scanning electron microscopy revealed the ball-milled SrFe_12_O_19_ particles to have sizes varying over several hundred nanometers. X-Ray powder-diffraction studies performed on the ball-milled particles before sintering clearly demonstrate the occurrence of a pronounced amorphization process. During sintering at 950 ^o^C, re-crystallization takes place, even for short sintering times of only 2 minutes and transformation of the amorphous phase into a secondary phase is unavoidable. The concentration of this secondary phase increases with increasing ball-milling time. The remanence and maximum magnetization values at 1T are weakly influenced, while the coercivity drops dramatically from 2340 Oe to 1100 Oe for the consolidated sample containing the largest amount of secondary phase.

SrFe_12_O_19_ (SFO) is a well-known synthetic magnetic material, which possesses an M-type hexaferrite structure similar to that of magnetoplumbite, a natural magnetic material. It was developed in the 1950s by scientists at Philips Laboratories and since then manufactured in large-scale production for permanent magnets with respectable magnetic properties at room temperature and a maximum energy product in the range 28–34 kJ/m^3^ for anisotropic magnets. Although the maximum energy product of this permanent magnet is about 15 times lower than the best permanent magnets on the market nowadays, SrFe_12_O_19_ is still attracting attention as it shows good thermal/chemical stability, a moderate value of magnetization^1^, a high Curie temperature^2^ and low production costs. Moreover, due to the character of the magnetocrystalline anisotropy, with its easy axis of magnetization along the *c*-direction, SrFe_12_O_19_ particles provide the opportunity to optimize specific parameters, such as anisotropy, remanent magnetization or coercive field, which are strongly dependent on the microstructure, sintered density and size of the constituent particles, where, *e*.*g*., a single magnetic domain structure is required for enhanced coercive fields^3^.

In order to produce a material consisting of a single-domain structure different synthesis routes and methods were considered throughout time. Most of these routes involve a low-temperature solid-state reaction^4^, hydrothermal synthesis^5^, sol-gel^6^, co-precipitation^7^,^8^,^9^, micro-emulsion^10^ or mechano-chemical synthesis^11^,^12^ and are mainly focused on the reduction of the particle size down to the nanoscale level where the formation of a magnetic multi-domain structure can be prevented.

In this work we have focused on reducing the particle size of a commercially available starting material using ball milling. The aim is to increase the coercive field by the reduction of the particle size to single-domain particles. The main purpose of this study was to find a direct correlation between the milling conditions and the magnetic properties upon consolidation using spark-plasma sintering (SPS). To our knowledge, this is the first reported study where SPS was used to sinter SrFe_12_O_19_ in a bulk form. Due to the uniaxial pressure as well as the applied DC current, SPS is a suitable method for obtaining a material with good mechanical strength and minimal grain growth due to the very short sintering times.

## Results

**Figure 1:**
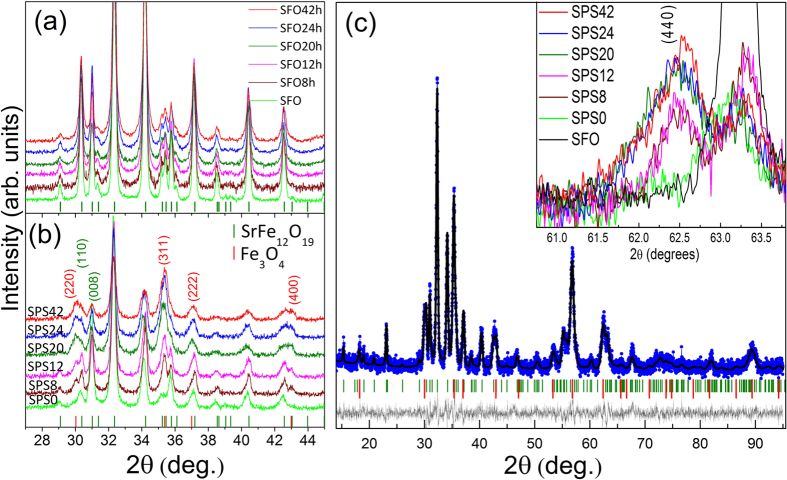
X-ray structural characterization. (**a**) The XRD patterns of all investigated powders before the SPS process. (**b**) Patterns after the SPS process. (**c**) A typical powder-diffraction pattern (blue data points) recorded on a SPS consolidated ball-milled powder (24 hours) together with the calculated pattern after Rietveld refinement (the black line). The grey line indicates the difference between the observed and calculated patterns. The inset plot gives a comparison between the starting SFO powder pattern and patterns after SPS consolidation. The detected (004) Bragg reflection corresponds to the secondary phase, Fe_3_O_4_.

**Figure 2:**
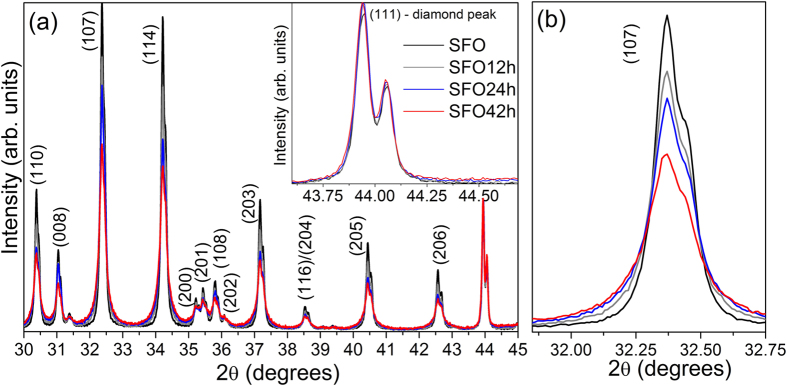
Crystallinity. (**a**) X-ray diffractograms of SrFe_12_O_19_ for ball-milled and un-milled powders with the addition of 30 weight% internal standard (diamond). The inset shows the (111) peak corresponding to the internal standard phase. (**b**) evolution of (107) peak with milling time corresponding to the SrFe_12_O_19_ phase.

X-ray powder diffraction investigations provide information about the crystal structure and phase purity of commercially un-milled/milled SrFe_12_O_19_ powders, before and after the SPS consolidation. Before SPS all the samples appear to be single phase, independent of milling time as seen in Fig. 1a. However, after SPS consolidation an additional phase was detected as observed in Fig. 2b. Based on a standard X-ray database (JCPDS), the secondary phase could be assigned to magnetite Fe_3_O_4_ (JCPDS number 00-019-0629), as determined from the lattice parameter, which is closer to 8.41 Å, than the maghemite (~8.34 Å)^13^. Refining the powder-diffraction pattern using SrFe_12_O_19_ and Fe_3_O_4_ results in a satisfactory fit of the data, as illustrated for SPS24 in Fig. 1c. The fitted R-values are shown in Table 1.

The SrFe_12_O_19_ was modeled using the space group P6_3_/mmc, while the Fe_3_O_4_ was described in Fd-3m. A March-Dollase model was included for handling the preferred orientation of SrFe_12_O_19_ along the (*00l*)-Bragg direction. The background was refined by a linear interpolation between a finite numbers of background points, while the peak width was modeled with a size and a microstrain parameter (Gaussian (I_G_) and Lorentian (X)). The lattice parameters and scale factors of the two phases were refined. All the other parameters, such as the position of the atoms, thermal parameters and site occupancy were kept fixed during the refinement. The powder X-ray diffraction (PXRD) data comparison between the diffractograms of the starting SrFe_12_O_19_ powder and the SPS consolidated powders in the vicinity of 2θ = 62.5^o^ is shown in the inset of Fig. 1. A quantitative analysis of the two phases, SrFe_12_O_19_ and Fe_3_O_4_, are represented in Table 1 together with their reliability factors. An increasing amount of Fe_3_O_4_ is detected when the powder used for the consolidation has spent a longer time in the planetary ball mill. The presence of SrO is virtually impossible to detect in the X-ray diffractograms as all the SrO peaks overlap with the SrFe_12_O_19_ and Fe_3_O_4_ phases. Therefore, the possible presence of SrO is not taken into account.

Table 1 contains information about the relative density of the pellets after the SPS compaction, which was calculated based on the geometry (*ρ* = *m*/*V*) and the theoretical density calculated from the Rietveld refinement. The final theoretical density, *ρ*_f_ was then recalculated according to the weight fractions (*W*_*f*_) of the two phases detected in each SPS sample using the following expression:


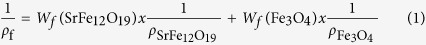


All the compacted samples possess a good compaction density, i.e., higher than 90%. The SPS consolidation was performed under similar conditions for all the investigated samples, implying that the differences observed in the powder-diffraction patterns are due to the variation in the milling time. To further investigate this feature, four samples were selected for a crystallinity test: the starting material and samples milled for 12, 24 and 42 hours. Figure 2 presents the PXRD patterns of the SrFe_12_O_19_ powders before and after the ball-milling mixed with an internal standard. From the inset of Fig. 2a it is clear that the diamond does not overlap with any peaks from SrFe_12_O_19_ and it is easy to distinguish between the two phases.

Diamond was chosen because of the low X-ray absorption, its absolute crystallinity and small particles size (~1 μm), which reduces micro-absorption effects^14^. The close-up of the (107) reflection given in Fig. 2b clearly illustrates how the peak corresponding to the SrFe_12_O_19_ phase is strongly affected by increasing the milling time. The peak profiles are changing with longer milling times; the peak intensities decrease and the peak broadens at the base, suggesting a decrease in the crystallite size and increase of strain. This feature is observed for all the reflections corresponding to the SrFe_12_O_19_ phase. On the other hand, an examination of the peak profiles shown in the inset of Fig. 2a reveals that the (111) peak reflection corresponding to the diamond does not change its intensity and remains constant for all the investigated samples. This observation supports the idea that a homogeneous mixture of the diamond and hexaferrite was obtained for all four samples and the reduced intensities observed in Fig. 2a are caused by a decrease in the crystallinity. A preferred orientation, which would cause a difference in the peak intensities, can be excluded as all the peaks from the different (*hkl*) of the planes show the same trend. Based on a Rietveld refinement the weight fractions of diamond and SrFe_12_O_19_ were determined and the sample crystallinity was calculated based on the following formula:


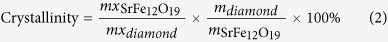


where 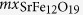 and *mx*_*diamond*_ represent the masses in percentage extracted from the Rietveld refinement, while *m*_*diamond*_ and 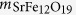 are the known masses obtained by weighing before mixing.

**Figure 3:**
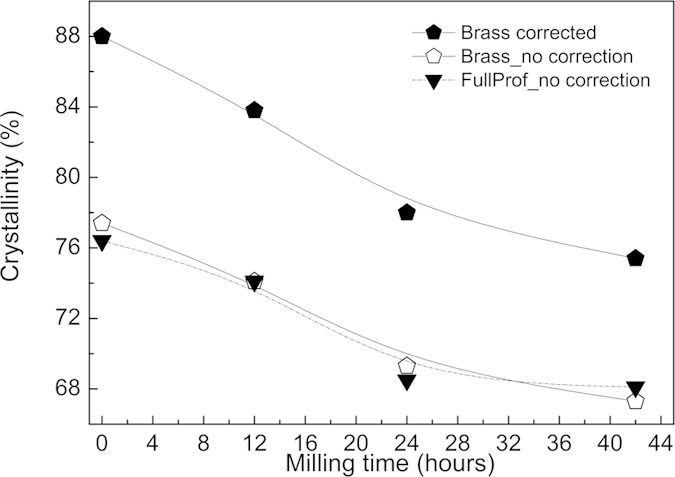
Crystallinity results. Crystallinity evolution of the SrFe_12_O_19_ function of the milling time calculated based on equation (2). Results obtained with and without the Brindley micro-absorption correction. The uncertainties caused by the sample preparation and the data collection are estimated to be 5%. The lines are a guide to the eye.

**Figure 4:**
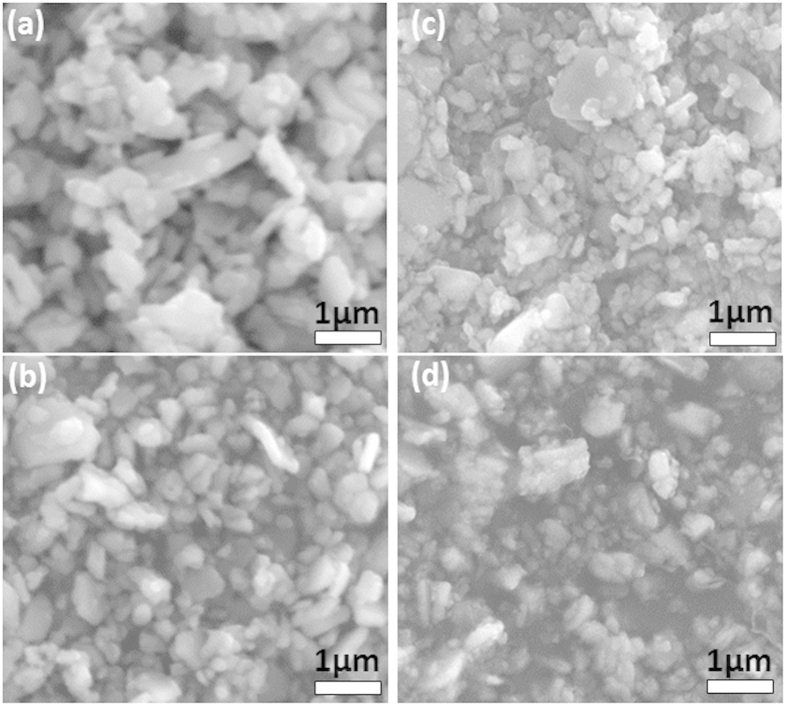
SEM analysis. (**a**) Micrograph of the starting powder. Powders with different milling time: (**b**) SFO12h, (**c**) SFO24h, (**d**) SFO42h.

The degree of crystallinity for various milling time is shown in Fig. 3 and was calculated based on equation

(2), where two software packages, *FullProf* and BRASS 2.0^15^, were used to extract 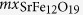 and *mx*_*diamond*_. In BRASS 2.0 the Brindley microabsorption-correction method is included as a post-refinement procedure taking the absorption factors and particle size into account. In this case the particles radii were obtained from the SEM images and the absorption for the Cu radiation was calculated to be *μ*(SrFe_12_O_19_) = 1049 cm^−1^ and *μ*(diamond) = 16.2 cm^−1^. The Rietveld analysis without correction gives similar results for both packages, while the Brindley correction causes the crystallinity to increase by about 10%. Although the absolute values may vary, it is clear that a continuous decrease in the crystallinity is observed for powders with a longer milling time. These results reveal that ball milling has a negative influence on the degree of the crystallinity, with a continuous decrease down to, *e*.*g*., 78%, for the sample being treated for the longest time (42 h) in the ball mill. It is also worth pointing out that the initial commercial powder used in this study (SrFe_12_O_19_) has a crystallinity of 88%. The morphological information about the SrFe_12_O_19_ particle size before and after the ball milling was obtained from the SEM and four representative micrographs are shown in Fig. 4a–d. The image in Fig. 4a, obtained from the initial SrFe_12_O_19_ powder, reveals irregular particle shapes with a large particle size distribution.

The average particle size was estimated using the ImageJ software by counting more than 100 particles for each investigated powder. In the case of the SrFe_12_O_19_ starting powder, particles with a size in the range 400–2200 nm were observed, with an average size of 640 nm. The size distribution observed for the initial particles suggests that the powder was milled, which is in line with the observed crystallinity (~88%). The facets observed in Fig. 4a, point to a post heat treatment after ball milling. The evolution of the average particle size with the milling time is given in the last column of Table 1. A decrease in the average particle size down to 400 nm was achieved with the longest milling time. A large size distribution is still present even after the longest milling time, with observed particles size up to 1.1 μm. Moreover, a longer ball-milling time leads to small aggregates, as seen in Fig. 4c,d. Therefore, using ball milling to decrease the particle size and obtain homogeneous nanopowders is challenging for SrFe_12_O_19_.

**Figure 5:**
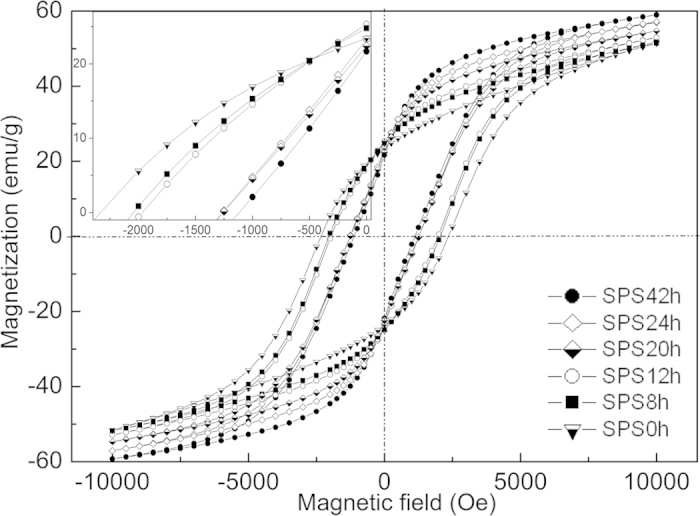
Magnetization results. Hysteresis loops taken on samples produced by SPS. The inset plot gives a close-up of the second quadrant of the hysteresis loops.

**Figure 6:**
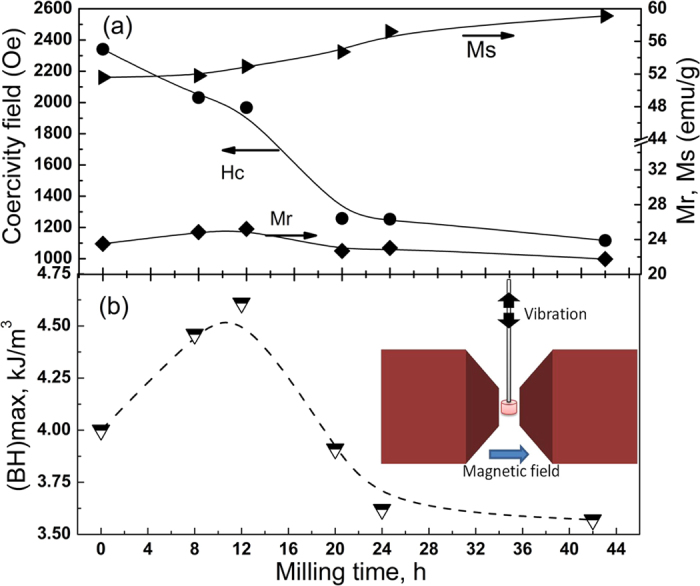
Maximum energy product. (**a**) Evolution of magnetic parameters subtracted from hysteresis loops shown in Fig. 5b, (**b**) Calculated maximum energy product values for different milling times, after SPS. The external magnetic field was applied perpendicular to the uniaxial SPS pressing direction.

Figure 5 shows the magnetic hysteresis loops of the SrFe_12_O_19_ after milling and consolidation by SPS. The hysteresis loops exhibit magnetic properties characteristic of a hard magnetic material. The magnetic parameters, such as coercivity (*H*_*c*_), remanent magnetization (*M*_*r*_) and maximum magnetization values at 1T (*M*_*s*_), were extracted from the hysteresis loops and their evolution with milling time plotted in Fig. 6a. Two different features can be distinguished, i.e., a pronounced decrease of *H*_*c*_ from 2349 Oe down to 1200 Oe and an increase of *M*_*s*_ from 50 emu/g up to 60 emu/g, for powders that were ball milled for a longer time. The coercivity is expected to increase with a decreasing particle size, especially when reaching a mono-domain state^16^. In the case of SrFe_12_O_19_ it was shown that the transition from single-domain to multi-domain structures is below 500 nm^17^,^18^. According to the SEM results, we should be below this transition size after an extended milling time of 20 hours. The fact that the opposite behavior is observed could indicate that the bulk properties are largely conditioned by the extrinsic properties of the secondary phase, Fe_3_O_4_, formed after the SPS, rather than a particles-size-reduction effect. For example, the highest coercivity of *H*_*c*_ = 2349 Oe was obtained for the sample SPS0, which contains the smallest amount of Fe_3_O_4_. As the amount of Fe_3_O_4_ is increased, the *H*_*c*_ decreases down to 1100 Oe for the SPS42 sample. This dilution effect is due to the Fe_3_O_4_ possessing much smaller values of *H*_*c*_ compared to SrFe_12_O_19_ phase^19^. However, another effect that could cause the continuous decrease of *H*_*c*_ may be related to a decrease of the shape anisotropy induced with longer milling time. It was experimentally observed in SrFe_12_O_19_ nanofibers, that a large length to diameter ratio leads to an enhancement of coercivity field and vice versa^20^.

The increase of *M*_*s*_ is most likely to be associated with the increased amount of magnetite phase, as in bulk form it possesses larger saturation magnetization values^21^,^22^ of about 90 emu/g, compared with strontium hexaferrite^23^,^24^, which is close to 75 emu/g. The continuous increase of magnetization up to 1T and the non-saturating behavior observed in all samples could be a signature of crystallite alignment. According to the PXRD data shown in Fig. 1b the SPS pressed samples have some preferred orientation along (*00l*) *i.e.* the easy axis of magnetization is perpendicular to the pellet surface. In the VSM measurements the magnetic field is applied perpendicular to the crystallite alignment, the setup is shown in Fig. 6b (insert). The misalignment between the easy axis and applied magnetic field is the reason why the saturation magnetization is not reached with the 1 T applied field.

The maximum energy product (BH)_max_, which is defined as the maximum area defined by a rectangle in the second quadrant of the hysteresis curve was calculated and shown in Fig. 6b. To calculate (BH)_max_ a correction for the self-demagnetization effects has been approximated by using a demagnetizing factor of 0.33, a value typically used for bonded/bulk magnets^25^. The (BH)_max_ values were found to be in the range 3.5–4.6 kJ/m^3^, with the highest values obtained for the samples with the shorter milling times, SPS8 and SPS12. These values are in good agreement with previous studies performed on ball-milled SrFe_12_O_19_. Nevertheless, enhanced values up to 9.6 kJ/m^3^ were reported as the milled powders were subjected to a post-annealing process^26^. The improvement in the magnetic properties is generated by the crystallization of the amorphous fraction. The atmosphere in which the post-annealing process is performed plays a very important role in the crystallization process. While annealing in air at 750–1000 ^°^C leads to the crystallization of small particles of pure strontium hexaferrite^27^, heating under vacuum conditions promotes the formation of magnetite as a secondary phase^28^,^29^,^30^. These observations coincide with our findings, where sintering by SPS under vacuum conditions led to the formation of magnetite, as confirmed by our PXRD investigations.

## Conclusion

The ball milling of commercial strontium hexaferrite powder for up to 42 h allows a particle size reduction down to 400 nm. Conventional PXRD indicates that the ball-milled samples were single phase, with no traces of impurities. However, the crystallinity is reduced continuously as the milling time is prolonged, down to 78% for the sample ball-milled for 42 hours. A very short sintering process (2 minutes) performed by SPS at 950 °C leads to the formation of an additional crystalline phase (Fe_3_O_4_), which increases its concentration up to 29% when consolidating particles milled for 42 hours. The resulting materials reveal changes in their magnetic behavior, with an increased maximum magnetization at 1T associated with a larger amount of secondary phase, but a decrease in the coercivity and remanent magnetization. The best obtained energy products are in the range 4.0–4.6 kJ/m^3^ for the samples with the lowest Fe_3_O_4_ content.

## Methods

### Preparation of SrFe_12_O_19_ by ball milling

The initial strontium hexaferrite powder used in this study was purchased from Iskra Feriti Co. (strength class 12.7–13.5 kJ/m^3^ and purity 99%). This powder was subjected to a grinding process performed at room temperature and at an ambient pressure in atmospheric air. The ball milling was done with a Planetary Mill PULVERISETTE 5 classic line with two grinding-bowl fasteners and a revolution speed of 150 rpm. A ball-to-powder ratio of 6:1 was used with 35 ml of ethanol serving as a dispersion medium. Under these milling conditions, five different powders were obtained by varying the milling time: 8, 12, 20, 24 and 42 hours. A summary of the milling conditions, together with an identification name for each sample studied in this work, is given in Table 1.

### SrFe_12_O_19_ consolidation by spark-plasma sintering

The consolidation of all the investigated powders was performed under vacuum, under similar conditions, using a SPS apparatus, model Dr. Sinter 511S/515S. A sample mass of 0.5 g was loaded in a graphite die with an 8-mm inner diameter. From our pre-investigations of the SPS conditions (not shown in this work) we could identify the optimum consolidation parameters to obtain a material with a density of 4.9 g/cm^3^, a good mechanical strength and a minimal grain growth. Consequently, all the milled powders were prepared at a constant applied pressure of 80 MPa and a heating rate of 90 °C/min. The sintering was carried out at 950 °C, with a holding time of 2 minutes, followed by free cooling. The as-obtained pellets have a diameter of 8 mm and a typical thickness of 2 mm. The pellet was polished to remove any traces of carbon paper used in the consolidation process. An identification name is given in the first column of Table 1 for each SPS consolidated material.

### Characterizations

#### X-ray powder diffraction

The structural characterizations of the milled powders and the SPS-consolidated pellets were performed by X-ray diffraction (PXRD) using a Rigaku diffractometer equipped with a rotating Cu anode and cross-beam optics selection Cu-K_α_ (*λ*_α1_ = 1.54059 Å, *λ*_α2_ = 1.54441 Å). The data were collected in the angular 2θ range 14° < 2θ < 95° using a D/tex Ultra detector running in the fluorescence-suppression mode. The pressed pellet was mounted with the flat surface towards the incoming beam and during the measurement the sample was spun in-plane to enhance the powder averaging. A quantitative phase analysis was carried out using Rietveld refinements with the program suite FullProf^31^. Instrumental line broadening was handled by an instrumental resolution file (.irf) created based on data collection under identical conditions of a LaB_6_ standard NIST 660b. Additional experiments were performed to determine the crystallinity, due to a suspected amorphization process. Crystallinity measurements were performed by adding 30 weight % of fully crystalline diamond powder to the ball-milled powders. The powders were dispersed manually using an agate mortar.

#### Scanning electron microscopy

Information about the particles size and morphology of commercial and ball-milled samples were obtained from scanning electron microscopy (SEM) investigations using a JEOL JSM 5800 microscope equipped with SE and BE detectors and an ISIS 300 EDS detector.

#### Vibrating sample magnetometry

Magnetic hysteresis loops for all the SPS-consolidated pellets were measured at room temperature in the presence of an applied external magnetic field of 1 Tesla using a MicroSense VSM EZ7 model vibrating-sample magnetometer. During the measurement the external magnetic field was applied perpendicular to the direction of the applied SPS uniaxial pressure.

## Additional Information

**How to cite this article**: Stingaciu, M. *et al.* Magnetic properties of ball-milled SrFe_12_O_19_ particles consolidated by Spark-Plasma Sintering. *Sci. Rep.* **5**, 14112; doi: 10.1038/srep14112 (2015).

## Figures and Tables

**Table 1:** Associated sample IDs used throughout the paper for powders after milling and after consolidation by SPS.

ID SPS sample	Initial powders used for SPS	Milling time (hours)	SrFe_12_O_19_(P6_3_/mmc)		Whole pattern parameters		Fe_3_O_4_ (Fd-3m)		Compaction density (%)	SEM average particle size, before SPS (nm)
			Weight fraction (%)	Bragg factor, R_B_ (%)	Rp (%)	Rwp (%)	Weight fraction (%)	Bragg factor, R_B_(%)		
SPS0	SFO	0	96(1)	11.9	7.8	10.1	4(1)	14.6	97(2)	6.4(1.9)·10^2^
SPS8	SFO8h	8	87(1)	7.5	6.4	8.2	13(1)	7.1	96(2)	5.8(1.8)·10^2^
SPS12	SFO12h	12	86(1)	8.1	6.6	8.4	14(1)	4.9	92(2)	5.4(1.6)·10^2^
SPS20	SFO20h	20	76(1)	7.4	6.4	8.2	24(1)	7.2	92(2)	5.0(1.1)·10^2^
SPS24	SFO24h	24	75(1)	7.0	6.6	8.3	25(1)	7.7	94(1)	4.7(0.9)·10^2^
SPS42	SFO42h	42	71(1)	7.3	7.0	8.8	29(1)	6.9	95(2)	4.0(0.8)·10^2^

Quantitative data extracted from the PXRD Rietveld refinement performed on the SPS pellets. The average particle size determined by the SEM is given in the last column.
